# Preclinical Evaluation of a New Format of ^68^Ga- and ^111^In-Labeled Affibody Molecule Z_IGF-1R:4551_ for the Visualization of IGF-1R Expression in Malignant Tumors Using PET and SPECT

**DOI:** 10.3390/pharmaceutics14071475

**Published:** 2022-07-15

**Authors:** Yongsheng Liu, Shengze Yu, Tianqi Xu, Vitalina Bodenko, Anna Orlova, Maryam Oroujeni, Sara S. Rinne, Vladimir Tolmachev, Anzhelika Vorobyeva, Torbjörn Gräslund

**Affiliations:** 1 Department of Immunology, Genetics and Pathology, Uppsala University, 75237 Uppsala, Sweden; yongsheng.liu@igp.uu.se (Y.L.); tianqi.xu@igp.uu.se (T.X.); maryam.oroujeni@igp.uu.se (M.O.); anzhelika.vorobyeva@igp.uu.se (A.V.); 2 Department of Protein Science, KTH Royal Institute of Technology, 10044 Stockholm, Sweden; shyu5201@gmail.com; 3 Research Centrum for Oncotheranostics, Research School of Chemistry and Applied Biomedical Sciences, Tomsk Polytechnic University, 634050 Tomsk, Russia; bodenkovitalina@gmail.com (V.B.); anna.orlova@ilk.uu.se (A.O.); 4 Department of Medicinal Chemistry, Uppsala University, 75123 Uppsala, Sweden; sara.rinne@ilk.uu.se; 5 Affibody AB, 17165 Solna, Sweden

**Keywords:** IGF-1R, PET, SPECT, gallium-68, indium-111, affibody molecules

## Abstract

The Insulin-like growth factor-1 receptor (IGF-1R) is a molecular target for several monoclonal antibodies undergoing clinical evaluation as anticancer therapeutics. The non-invasive detection of IGF-1R expression in tumors might enable stratification of patients for specific treatment and improve the outcome of both clinical trials and routine treatment. The affibody molecule Z_IGF-1R:4551_ binds specifically to IGF-1R with subnanomolar affinity. The goal of this study was to evaluate the ^68^Ga and ^111^In-labeled affibody construct NODAGA-(HE)_3_-Z_IGF-1R:4551_ for the imaging of IGF-1R expression, using PET and SPECT. The labeling was efficient and provided stable coupling of both radionuclides. The two imaging probes, [^68^Ga]Ga-NODAGA-(HE)_3_-Z_IGF-1R:4551_ and [^111^In]In-NODAGA-(HE)_3_-Z_IGF-1R:4551_, demonstrated specific binding to IGF-1R-expressing human cancer cell lines in vitro and to IGF-1R-expressing xenografts in mice. Preclinical PET and SPECT/CT imaging demonstrated visualization of IGF-1R-expressing xenografts already one hour after injection. The tumor-to-blood ratios at 3 h after injection were 7.8 ± 0.2 and 8.0 ± 0.6 for [^68^Ga]Ga-NODAGA-(HE)_3_-Z_IGF-1R:4551_ and [^111^In]In-NODAGA-(HE)_3_-Z_IGF-1R:4551_, respectively. In conclusion, a molecular design of the Z_IGF-1R:4551_ affibody molecule, including placement of a (HE)_3_-tag on the N-terminus and site-specific coupling of a NODAGA chelator on the C-terminus, provides a tracer with improved imaging properties for visualization of IGF-1R in malignant tumors, using PET and SPECT.

## 1. Introduction

Insulin-like growth factor-1 receptor (IGF-1R) overexpression is involved in the neoplastic transformation of cells, contributing to a malignant phenotype by an enhanced proliferation rate and suppressed apoptosis [1,2]. The involvement of IGF-1R overexpression in the development, progression, metastasis, and therapy resistance of several malignancies, such as breast [3,4], prostate [5,6], pancreatic [7], and ovarian [8] cancers, is well documented in both preclinical and clinical studies. Understanding the importance of IGF-1R signaling has prompted the development and clinical evaluation of several potential anticancer therapeutics, targeting signaling through the IGF axis [9]. These drug candidates are, for example, monoclonal antibodies, directly interacting with IGF-1R, and they include ganitumab [10,11,12], figitumumab [13,14], cixutumumab [15,16], and dalotuzumab [17,18]. The results from clinical trials including these mAbs has unfortunately only shown a modest clinical benefit for the patients. However, the clinical trials were performed on unselected patient groups, and a more prominent response was found for a subset of the ovarian [10], pancreatic [19], and prostate [20] cancer patients. Moreover, the combination of the standard regimen with such antibodies was sometimes associated with an increased rate of adverse effects [11,13]. It has therefore been suggested that a subset of patients might benefit from IGF-1R-targeted therapy on the precondition of identification of the appropriate predictive biomarkers [11,21,22]. Both preclinical and clinical data suggest that the antiproliferative effect of anti-IGF-1R antibodies correlates with the level of IGF-1R expression [23,24,25]. Accordingly, identification of the tumors overexpressing IGF-1R might enable stratification of the patients that would benefit the most from such therapies and help to harness the full potential of IGF-1R-targeting pharmaceutics.

The most straightforward approach to measure the expression level would be to analyze biopsy samples. However, the invasive nature of biopsies limits the number of samples that can be collected and does not address the possible heterogeneity of expression of IGF-1R in the primary tumor and the metastases, or the variation of the expression over time. Apparently, the development of a non-invasive methodology for the determination of the IGF-1R status would allow for repeated measurements and would facilitate both clinical development and the routine use of IGF-1R-targeted pharmaceutics. A possible solution might be PET (positron emission tomography) or SPECT (single photon emission computed tomography) visualization of the expression by radiopharmaceuticals specifically binding to IGF-1R in vivo.

The use of radionuclide visualization of molecular target expression for subsequent radionuclide therapy (theranostics) is an established practice in the treatment of thyroid, prostate, neuroendocrine, and hematologic malignancies [26]. Currently, theranostic approaches are also finding use in regimens including monoclonal antibodies and antibody-drug conjugates [27]. In the case of IGF-1R, the development of imaging probes is challenging because of its noticeable expression in a number of healthy tissues, e.g., the lung, liver, intestines, and salivary gland (Human Protein Atlas: https://www.proteinatlas.org/ENSG00000140443-IGF1R/tissue, accessed on 15 June 2022). Furthermore, the overexpression in tumors is often modest, and only a relatively low expression level (10,000–30,000 receptors per cell) is necessary for the tumor to respond to IGF-1R-targeting antibodies [23,24]. This puts high demands on the imaging properties of the probes for IGF-1R visualization.

Several classes of radiolabeled probes for visualization of IGF-1R have been preclinically evaluated [28]. For example, the small-molecule IGF-1R tyrosine kinase inhibitor BMS-754807 was radiofluorinated with an aim to be used for PET imaging [29]. [^18^F]F-BMS-754807 demonstrated specific binding to glioblastoma, breast cancer, and pancreatic tumor sections in vitro [29], and its biodistribution was evaluated in rodents [30]. However, no tumor imaging using [^18^F]F-BMS-754807 has yet been reported.

Radiolabeled therapeutic anti-IGF-1R antibodies have been evaluated for imaging of IGF-1R in preclinical models [31,32,33]. R1507, a fully human monoclonal antibody against IGF-1R, has been labeled with ^111^In for SPECT, using benzyl-isothiocyanate-diethylenetriaminepentaacetic acid (DTPA). It has been shown that the uptake of [^111^In]In-R1507 in tumors correlates with the IGF-1R expression level [31]. However, the contrast of imaging was low due to the long residence time of IgG in blood, a consequence of its large size (molecular weight 150 kDa), and interaction with the neonatal Fc receptor (FcRn), which protects IgG from degradation by cells in contact with blood [34]. Seven days were required to reach a tumor-to-blood ratio of 8.1 ± 2.5 for tumors with a high expression level of IGF-1R. To utilize the imaging power of PET, the long-lived positron emitter ^89^Zr was used to label another anti-IGF-1R antibody, 1A2G11 [33]. In this case, the tumor-to-blood ratio was around 1 even 120 h after injection.

The use of smaller imaging agents improves the contrast [35]. Indeed, the use of a smaller F(ab’)_2_-fragment (molecular weight of 110 kDa, and lacking interaction with FcRn) of the R1507 antibody resulted in a significantly increased tumor-to-blood ratio [32,36]. A tumor-to-blood ratio of 7.7 was achieved for a SUM149 breast cancer xenograft mouse model at 24 h after injection [32]. This indicated that the use of smaller protein-based imaging probes might improve the IGF-1R imaging contrast. IGF-1(E3R), a synthetic analogue of a natural ligand IGF-1, which is not recognized by IGF binding protein-3 (IGFBP-3), has been conjugated with DTPA by using its cyclic anhydride and labeled with ^111^In [37]. This small (7.5 kDa) tracer demonstrated a strong correlation between its uptake and the IGF-1R expression level in human tumor xenografts in mice. The uptake in tumors with high IGF-1R expression (MCF-7/HER2-18) was 2.5 ± 0.4% ID/g, and the tumor-to-blood ratio was 5.8 at 4 h after injection. A possible disadvantage of this tracer was the stochastic attachment of the label due to conjugation of the chelator to any one of the four amino groups in the protein. Thus, the labeled product was a mixture of several variants with different positions of the chelator, which might affect the biodistribution and performance of the tracer.

**Figure 1:**
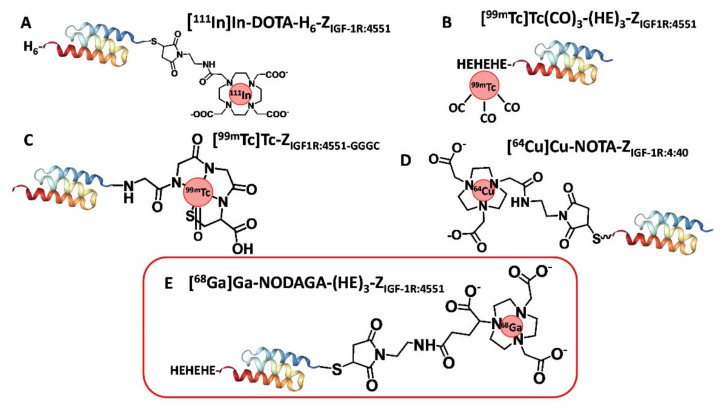
Structures of affibody molecules, which were evaluated for imaging of IGF-1R expression. (**A**) ^111^In]In-DOTA-H_6_-Z_IGF-1R:4551_, (**B**) [^99m^Tc]Tc(CO)_3_-(HE)_3_-Z_IGF-1R:4551_, (**C**) [^99m^Tc]Tc-Z_IGF-1R:4551_-GGGC, (**D**) ^64^Cu-NOTA-Z_IGF-1R:4:40_, (**E**) [^68^Ga]Ga-NODAGA-(HE)_3_-Z_IGF-1R:4551_. The affibody molecule [^68^Ga]Ga-NODAGA-(HE)_3_-Z_IGF-1R:4551_ (evaluated in this study) is marked with a red frame.

A possible alternative way to develop small probes for molecular imaging of IGF-1R is to use affibody molecules. They are a class of small proteins (molecular weight of approximately 6.5 kDa), based on a non-immunoglobulin triple-helical scaffold [38]. Affibody molecules with strong affinity and high specificity to a variety of cancer-associated molecular targets have been isolated from combinatorial libraries by using different selection techniques [39]. For radionuclide imaging of IGF-1R in vivo, the affibody molecule Z_IGF-1R:4551_, which binds to IGF-1R expressing cells with the affinity (equilibrium dissociation constant, K_D_) 500 ± 79 pM, has been developed [40]. The feasibility of using Z_IGF-1R:4551_ for SPECT imaging of IGF-1R has been demonstrated in a mouse model, using [^111^In]In-DOTA-H_6_-Z_IGF-1R:4551_ (Figure 1A) [40]. In this tracer, a DOTA chelator was conjugated to a unique cysteine placed in the C-terminal end of the affibody construct, which enabled uniform labeling. For further development, a histidine–glutamate–histidine–glutamate–histidine–glutamate tag ((HE)_3_- or HEHEHE-tag) was introduced at the N-terminus of Z_IGF-1R:4551_, which could be utilized for labeling using [^99m^Tc]Tc(CO)_3_^+^ [41]. [^99m^Tc]Tc(CO)_3_-(HE)_3_-Z_IGF-1R:4551_ (Figure 1B) was shown to have a significantly better tumor-to-blood and tumor-to-liver ratio compared with [^111^In]In-DOTA-H_6_-Z_IGF-1R:4551_. Other studies have demonstrated that the use of a GGGC peptide-based chelator for the labeling of affibody molecules with technetium-99m enables a substantial reduction of the renal uptake [42]. Application of this approach to an IGF-1R targeting affibody molecule resulted in [^99m^Tc]Tc-Z_IGF-1R:4551_-GGGC (Figure 1C) [43]. The renal uptake of [^99m^Tc]Tc-Z_IGF1R:4551_-GGGC (7.5 ± 0.7% ID/g at 4 h) was substantially lower than the uptake of [^99m^Tc]Tc(CO)_3_-(HE)_3_-Z_IGF-1R:4551_ (135 ± 7% ID/g at 4 h). In addition, the tumor-to-blood ratio of [^99m^Tc]Tc-Z_IGF-1R:4551_-GGGC (6.2 ± 0.9 at 4 h) was higher than the ratios for [^111^In]In-DOTA-H_6_-Z_IGF-1R:4551_ (2.5 ± 0.2) or [^99m^Tc]Tc(CO)_3_-(HE)_3_-Z_IGF-1R:4551_ (3.5 ± 0.7) at the same time point in the same tumor model (DU145 prostate cancer xenografts in mice).

While SPECT cameras are widely available, PET imaging provides better spatial resolution and higher quantification accuracy. Therefore, the development of a PET imaging probe for IGF-1R would be desirable. For this purpose, Su and co-workers evaluated the affibody-based tracer NOTA-Z_IGF-1R:4:40_ labeled with a long-lived positron emitter, ^64^Cu (Figure 1D) [44]. This tracer provided a tumor-to-blood ratio of 4.1 ± 0.6 at 24 h after injection. Although the use of NOTA provides stable coupling of ^64^Cu [45], earlier studies on ^64^Cu-labeled anti-HER2 affibody molecules have shown that the renal metabolism of such tracers after reabsorption in the proximal tubuli causes a release of ^64^Cu into the blood stream, decreasing the tumor-to-blood ratio [46].

An alternative positron-emitting nuclide for labeling of anti-IGF-1R affibody molecules is ^68^Ga (T_1/2_ = 68 min). To investigate the performance of a ^68^Ga-labeled variant of Z_IGF-1R:4551_, a new design was applied in this study (Figure 1E). A (HE)_3_-tag was introduced at the N-terminus since it has previously been found that the use of this tag can favorably influence the biodistribution of affibody molecules [47]. For coupling of the radionuclide, a maleimido derivative of the macrocyclic triaza-chelator NODAGA (1,4,7-triazacyclononane-1-glutaric acid-4,7-diacetic acid) was conjugated to a unique cysteine placed in the C-terminus of the affibody construct. NODAGA provides stable chelation with ^68^Ga [48]. It has to be noted that it is desirable to have the possibility of SPECT imaging of IGF-1R expression, since SPECT scanners are more common than PET cameras in the medical community. The use of NODAGA in NODAGA-(HE)_3_-Z_IGF-1R:4551_ provides the possibility for labeling with the single-photon emitter ^111^In [49].

The goal of this study was to perform a preclinical evaluation of NODAGA-(HE)_3_-Z_IGF-1R:4551_, labeled with ^68^Ga or ^111^In, for imaging of IGF-1R expressing tumors by using PET and SPECT.

## 2. Materials and Methods

### 2.1. General

Most of the chemicals used in the study were purchased from Sigma-Aldrich Sweden (Stockholm, Sweden). The buffers used for labeling were prepared by using high-quality Milli-Q water and purified from metal contaminations, using a Chelex 100 resin (Bio-Rad Laboratories, Hercules, CA, USA). No-carrier-added ^111^InCl_3_ was purchased from Curium Pharma (Stockholm, Sweden). Gallium-68 was obtained by elution of a ^68^Ge/^68^Ga generator (Cyclotron Co. Obninsk, Russia) with 0.1 M HCl. Maleimido derivative of the chelator NODAGA (2,2′-(7-(1-carboxy-4-((2-(2,5-dioxo-2,5-dihydro-1H-pyrrol-1-yl)ethyl)amino)-4-oxobutyl)-1,4,7-triazonane-1,4-diyl)diacetic acid) was purchased from CheMatech (Dijon, France). A CR 35 Bio scanner and CR-reader Plus software (Raytest Isotopenmeßgeräte, Straubenhardt, Germany) were used for measuring the radioactivity distribution on the instant thin-layer chromatography (ITLC) strips.

The IGF-1R-expressing cell line DU145 (prostate cancer) and the ovarian cancer cell line SKOV3 were obtained from the American Type Culture Collection (ATCC). The cells were cultured in Roswell Park Memorial Institute (RPMI) 1640 medium (Sigma-Aldrich, St. Louis, MO, USA) supplemented with 10% fetal calf serum, 2 mM L-glutamine, 100 IU/mL penicillin, and 100 mg/mL streptomycin. The data on the in vitro studies and biodistribution were analyzed by using GraphPad Prism (version 8.0.1 for Windows; GraphPad Software, La Jolla, CA, USA) to determine significant differences (*p* < 0.05, *t*-test).

### 2.2. Protein Production

The gene encoding (HE)_3_-Z_IGF-1R:4551_ with a C-terminally placed cysteine was expressed from the pET-21a(+) plasmid (Novagen, Madison, WI, USA), under control of the T7-promoter, essentially as earlier described for a different affibody construct [50]. Protein production was carried out overnight at 25 °C, after which the cells were harvested by centrifugation and lysed in a French Press. After heat treatment at 70 °C for 10 min, the (HE)_3_-Z_IGF-1R:4551_ protein was purified by anion-exchange chromatography on a Q-sepharose column, followed by reversed-phase chromatography on a Resources RPC column (GE Healthcare, Uppsala, Sweden), using an ÄKTA system (GE Healthcare). The fractions containing (HE)_3_-Z_IGF-1R:4551_ were pooled and lyophilized.

### 2.3. NODAGA Conjugation

(HE)_3_-Z_IGF-1R:4551_ was dissolved to a concentration of 1 mg/mL in PBS buffer (1 mL). To reduce potentially oxidized cysteines, tris (2-carboxyethyl) phosphine (TCEP) was added to a final concentration of 5 mM, followed by incubation for 30 min at 37 °C. The pH was adjusted to 6.5, using a 1 M HCl solution, after which the NODAGA chelator (20 mM in DMSO) was added to a 3:1 (chelator:protein) ratio. The conjugation reaction was allowed to proceed for 16 h at room temperature. The reaction mixture was purified (aliquot by aliquot) by reversed-phase high-performance liquid chromatography (RP-HPLC) on Agilent 1200 HPLC system (Agilent Technologies, Santa Clara, CA, USA), using a Zorbax 300SB-C18 column (9.4 × 250 mm, 5 μm particle size, Agilent Technologies, Santa Clara, CA, USA) with a 20 min gradient of 20–65% B (A = 0.1% trifluoroacetic acid (TFA) in H_2_O; B = 0.1% TFA in CH_3_CN), at a flow rate of 1.5 mL/min. An analysis of the purified product was performed on the same system, using a Zorbax CB300-C18 column (4.6 × 150 mm, 3.5 μm particle size, (Agilent Technologies, Santa Clara, CA, USA) with a 30 min gradient of 20–65% B (A = 0.1% trifluoroacetic acid (TFA) in H_2_O; B = 0.1% TFA in CH_3_CN), at a flow rate of 0.8 mL/min. The retention time of NODAGA-(HE)_3_-Z_IGF-1R:4551_ was 19.4 min. Verification of the correct mass of NODAGA-(HE)_3_-Z_IGF-1R:4551_ was performed by using a 6520 Accurate-Mass Q-TOF LC/MS instrument (Agilent Technologies, Santa Clara, CA, USA).

The fractions containing NODAGA-(HE)_3_-Z_IGF-1R:4551_ were pooled, lyophilized, and stored at −20 °C until labeling and biologic evaluation. Immediately before the evaluation, NODAGA-(HE)_3_-Z_IGF-1R:4551_ was redissolved in PBS to a concentration 3.4 mg/mL. Aliquots containing 20 µg in 5.9 µL PBS were prepared and stored at −20 °C.

### 2.4. Radiolabeling

For labeling with Ga-68, an aliquot of 20 µg of NODAGA-(HE)_3_-Z_IGF-1R:4551_ in PBS was mixed with 100 µL of 1.25 M sodium acetate, pH 3.6. The mixture was incubated with 80 µL of gallium-68 eluate (29–30 MBq) at 50 °C for 15 min. Thereafter, the labeled compound was incubated with a 500-fold molar excess of EDTA (Ethylenediaminetetraacetic acid) for 5 min at 50 °C. After incubation, 1 µL samples were collected for measurement of the radiochemical yield using ITLC (see below). The labeled conjugates were purified by using NAP-5 columns (GE Healthcare) pre-equilibrated with PBS containing 1% BSA. The radiochemical purity of the conjugates was determined by ITLC.

To evaluate its stability, the purified conjugate was incubated with a 1000-fold excess of EDTA for 1 h at room temperature. The control samples were incubated in the same conditions but without the addition of EDTA. The protein-bound activity was analyzed by using ITLC. The stability test was performed in triplicates.

For labeling with In-111, an aliquot of 20 µg of NODAGA-(HE)_3_-Z_IGF-1R:4551_ in PBS was mixed with 50 µL of 0.2 M sodium acetate, pH 6.0. The mixture was incubated with 30 µL of ^111^In solution in 0.1 M HCl (10 MBq) at 60 °C for 30 min. Thereafter, the labeled compound was incubated with a 500-fold molar excess of EDTA for 5 min at 60 °C. After incubation, 1 µL samples were collected for measurement of the radiochemical yield, using ITLC (see below). No purification was required since the radiochemical purity was over 95%.

The stability under EDTA challenge was determined in the same way as for the ^68^Ga-labeled variant, but the incubation time was 6 h.

Instant thin-layer chromatography (ITLC) was performed by using glass microfiber chromatography paper impregnated with a silica gel (Agilent, Santa Clara, CA, USA). The ITLC strips were developed by 0.2 M citric acid, pH 2.0. In this system, the radiolabeled affibody molecules remain at the application point (R_f_ = 0.0), while free radiometals (both ^68^Ga and ^111^In) and their complexes with EDTA migrate with the solvent front (R_f_ = 1.0).

To validate the ITLC results, a radio-HPLC analysis was performed. An Elite LaChrom system (Hitachi, VWR, Darmstadt, Germany) consisting of an L-2130 pump, a UV detector (L-2400), and a radiation flow detector (Bioscan, Washington, DC, USA) coupled in series was used. The analysis was performed by using an analytical reversed-phase (RP) column (Phenomenex, Aschaffenburg, Germany; Luna^®^ 5 µm C18, 100 Å; 4.6 × 150 mm). The RP-HPLC conditions were as follows: A = 10 mM TFA/H_2_O; B = 10 mM TFA/acetonitrile; UV-detection at 214 nm; gradient elution, 0–25 min at 5 to 70% B, 25–28 min at 70 to 95% B, and 29–30 min at 5% B; and flow rate was 1.0 mL/min.

### 2.5. In Vitro Studies

The cells were seeded one day prior to the experiments in 3 cm petri dishes, with a density of 10^6^ cells/dish. Each experiment was performed in triplicate for each data point.

The binding specificity of [^68^Ga]Ga-NODAGA-(HE)_3_-Z_IGF-1R:4551_ and [^111^In]In-NODAGA-(HE)_3_-Z_IGF-1R:4551_ to DU145 and SKOV3 cells was tested by incubating the cells with 1 nM of labeled conjugates for 30 min at 37 °C. To saturate the IGF-1 receptors, the cells were incubated with a 1000-fold excess of unlabeled (HE)_3_-Z_IGF-1R:4551_ for 20 min before adding the labeled compound in one set of dishes. Afterward, the cells were washed and detached by trypsin, and the radioactivity in cells was measured by using an automated gamma-spectrometer with a NaI (TI) detector (2480 Wizard, Wallac, Finland) to calculate the percent of cell-bound radioactivity.

To study cellular processing of [^111^In]In-NODAGA-(HE)_3_-Z_IGF-1R:4551_, a modified acid wash method [51] was used. Briefly, DU145 and SKOV3 cells were continuously incubated with 3 nM labeled conjugate at 37 °C. At predetermined time points (1, 2, 4, 6, and 24 h after incubation start), a group of dishes (*n* = 3) was removed from the incubator, and the incubation medium was collected. To separate the membrane-bound radioactivity, the cells were treated with 0.2 M glycine buffer containing 4 M urea, pH 2.5, for 5 min on ice, and the solution was collected. To isolate the internalized fraction of the radioconjugates, the cells were detached by treatment with 1 M NaOH, at 37 °C, for 30 min and collected. The activities of the incubation medium, the acidic buffer containing the membrane-bound conjugate, and the cells with the internalized fraction were measured to determine the membrane-bound and the internalized fractions.

### 2.6. In Vivo Studies

The animal experiments were planned and performed in accordance with national legislation on laboratory animal protection, and the study was approved by the local Ethics Committee for Animal Research in Uppsala (Permit 4C/16).

To establish IGF-1R-positive and IGF-1R-negative xenografts, 5 × 10^6^ DU145 cells (in Matrigel, BD Biosciences) or Ramos cells (IGF-1R negative) were injected subcutaneously in the hind legs of female BALB/c *nu/nu* mice. The xenografts were allowed to grow for 2 weeks. In the biodistribution experiments, groups of four mice were used. At the time of the experiment, the average weight of mice with DU145 xenografts was 21.9 ± 1.0 g and 23.0 ± 0.3 g for mice bearing Ramos xenografts, respectively. The average tumor weight was 80 ± 37 mg and 46 ± 29 mg for mice bearing DU145 and Ramos xenografts, respectively.

The targeting properties of the ^111^In- and ^68^Ga-labeled NODAGA-(HE)_3_-Z_IGF-1R:4551_ conjugates were compared by injection of mixtures of both radiolabeled variants in the same mice. The time points for determination of the biodistribution were 1, 3, and 24 h p.i. for mice bearing DU145 xenografts. For measurement of the biodistribution at 1 h p.i., 220 kBq [^68^Ga]Ga-NODAGA-(HE)_3_-Z_IGF-1R:4551_ and 10 kBq ^111^In-labeled NODAGA-(HE)_3_-Z_IGF-1R:4551_ were mixed. For measurement of the biodistribution at 3 h p.i., 700 kBq ^68^Ga-labeled NODAGA-(HE)_3_-Z_IGF-1R:4551_ and 10 kBq ^111^In-labeled NODAGA-(HE)_3_-Z_IGF-1R:4551_ were used. For measurement of the biodistribution at 24 h after injection, only 40 kBq of ^111^In-labeled probe was used. The labeled conjugates were formulated for co-injection based on a total injected protein mass of 1 μg per mouse. At each time point, a group of mice was sacrificed by heart puncture after intraperitoneal injection of a mixture of ketamine (250 mg/kg) and xylazine (25 mg/kg). Samples of blood, salivary glands, lung, liver, spleen, pancreas, stomach, large intestine, kidneys, tumor, muscle, bone, and the remaining carcass were collected. Organs and tissue samples were weighed, and their activity was measured by using a gamma-spectrometer separately for ^68^Ga and ^111^In, as described earlier [52]. These values were used to calculate the uptake of ^111^In- and ^68^Ga-labeled NODAGA-(HE)_3_-Z_IGF-1R:4551_ as a percentage of injected dose per gram of tissue (%ID/g).

To test the in vivo specificity, a group of mice with IGF-1R-negative Ramos xenografts were injected with a mixture of 700 kBq [^68^Ga]Ga-NODAGA-(HE)_3_-Z_IGF-1R:4551_ and 10 kBq ^111^In-labeled NODAGA-(HE)_3_-Z_IGF-1R:4551_. Tumors and blood samples were collected at 1 h p.i., and their activity was measured as described above.

In vivo imaging was performed 1 h after injection to obtain a visual confirmation of the biodistribution data. Mice bearing DU145 xenografts were used for this purpose. One mouse was injected with 3.2 MBq (1 µg) [^68^Ga]Ga-NODAGA-(HE)_3_-Z_IGF-1R:4551_. Whole-body nanoPET images were acquired by using a nanoScan PET/MR (Mediso Medical Imaging Systems, Budapest, Hungary). The scan times were 45 min. A CT scan was performed immediately after the PET scan, using a nanoScan SPECT/CT (Mediso Medical Imaging Systems, Budapest, Hungary) with the same bed. The parameters for the CT scans were a 5 min acquisition time, an X-ray energy peak of 50 keV/670 μA, and 480 projections. A second mouse was injected with 1.2 MBq (1 µg) [^111^In]In-NODAGA-(HE)_3_-Z_IGF-1R:4551_. Whole-body SPECT/CT was performed by using nanoScan SPECT/CT (Mediso Medical Imaging Systems, Hungary). The acquisition time was 20 min. Gamma-peaks of 245 and 171 keV (window width of 20%) were used for acquisition. The CT scan was performed in the same way as in the case of [^68^Ga]Ga-NODAGA-(HE)_3_-Z_IGF-1R:4551_. A reconstruction of the scans was conducted by using the Tera-Tomo™ 3D reconstruction engine with decay correction at the injection administration time. The CT data were reconstructed by using filter back projection in Nucline 2.03 Software (Mediso Medical Imaging Systems Ltd., Budapest, Hungary). The PET and CT scans were fused by using InterView FUSION software (Mediso Medical Imaging Systems, Budapest, Hungary).

## 3. Results

### 3.1. Production, Purification, and Conjugation

**Figure 2:**
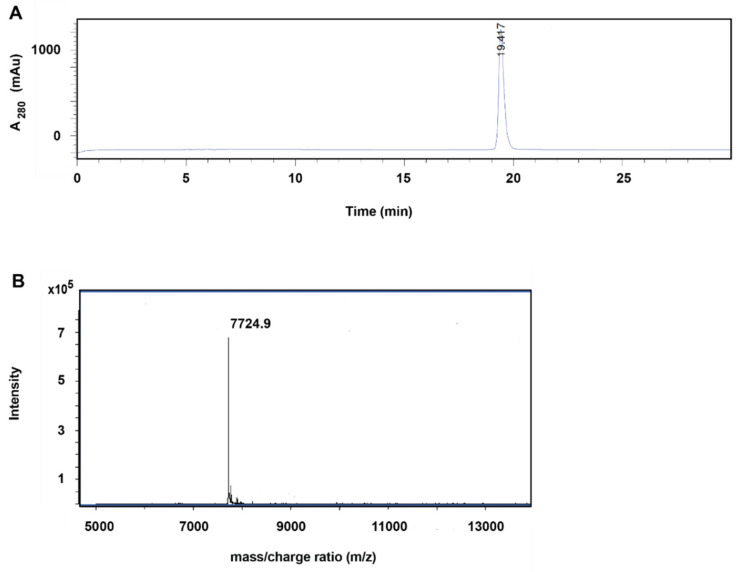
Analysis of NODAGA-(HE)_3_-Z_IGF-1R:4551_ by RP-HPLC (**A**) and mass spectrometry (**B**). The observed molecular weight was 7724.9 Da, and the calculated molecular weight was 7724 Da.

The (HE)_3_-Z_IGF-1R:4551_ protein was expressed in *Escherichia coli* and was purified by heat treatment, followed by anion-exchange and reversed-phase chromatography. The product was conjugated to NODAGA and thereafter purified by using reversed-phase chromatography. To analyze the purity of the conjugated protein, a sample was separated by analytical reversed-phase high-performance liquid chromatography (RP-HPLC, Figure 2A). The protein was eluted as a single symmetrical peak. Determination of the area-under-curve shows that the protein was >99% pure, close to 100%. The molecular weight was also determined by mass spectrometry (Figure 2B), and the result differed less than 1 Da from the theoretical molecular weight of NODAGA-(HE)_3_-Z_IGF-1R:4551_.

### 3.2. Radiolabeling

Labeling with ^68^Ga provided a radiochemical yield in the range of 84–96%. The radiochemical purity of [^68^Ga]Ga-NODAGA-(HE)_3_-Z_IGF-1R:4551_ after purification, using a size-exclusion NAP-5 column, was 96–97%. The specific activity of 1.5 MBq/µg (11.6 GBq/µmol) was reproducibly obtained for [^68^Ga]Ga-NODAGA-(HE)_3_-Z_IGF-1R:4551_. No measurable release was observed after the challenge with a large molar excess of EDTA (Table 1).

Labeling with ^111^In provided a radiochemical yield exceeding 98%, and no additional purification was required. The specific activity of 1.5 MBq/µg (11.6 GBq/µmol) was reproducibly obtained for [^111^In]In-NODAGA-(HE)_3_-Z_IGF-1R:4551_. In the EDTA challenge test, the difference between samples treated with EDTA and control samples was small and was within the accuracy of the method (Table 1).

**Figure 3:**
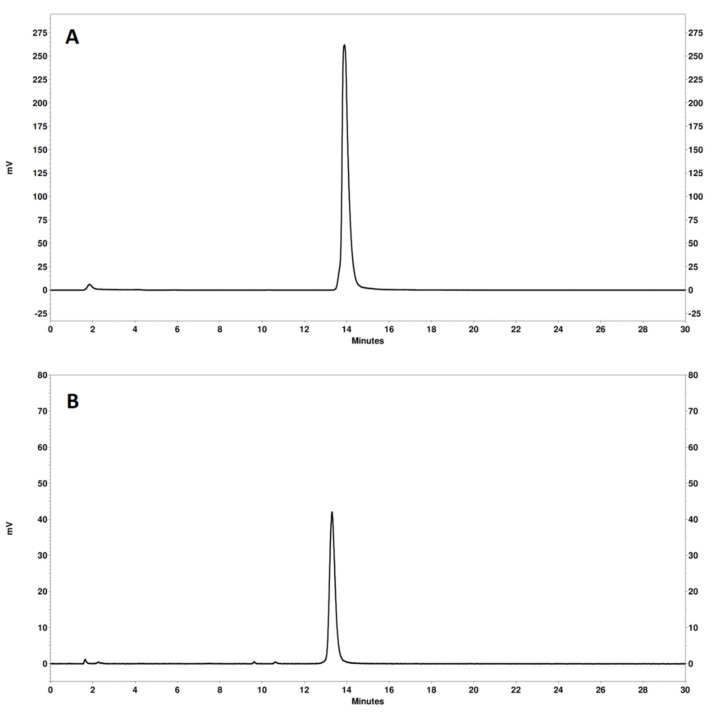
Representative radio-HPLC chromatograms of [^111^In]In-NODAGA-(HE)_3_-Z_IGF-1R:4551_ (**A**) and [^68^Ga]Ga-NODAGA-(HE)_3_-Z_IGF-1R:4551_ (**B**). The retention time of unlabeled NODAGA-(HE)_3_-Z_IGF-1R:4551_ (UV detection) was 13.2 min.

The radio-HPLC analysis (Figure 3) confirmed a high radiochemical purity of both [^68^Ga]Ga-NODAGA-(HE)_3_-Z_IGF-1R:4551_ and [^111^In]In-NODAGA-(HE)_3_-Z_IGF-1R:4551_.

### 3.3. In Vitro Studies

**Figure 4:**
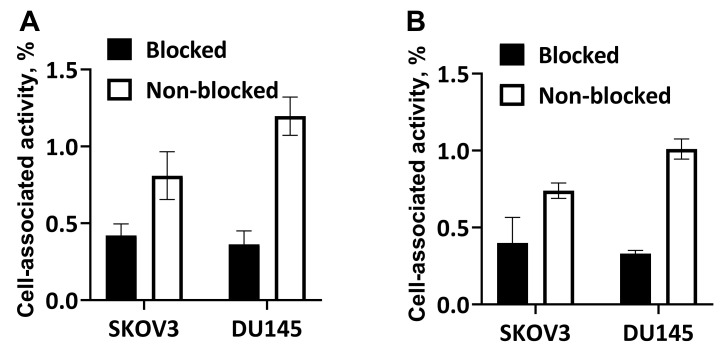
In vitro specificity of [^68^Ga]Ga-NODAGA-(HE)_3_-Z_IGF-1R:4551_ (**A**) and [^111^In]In-NODAGA-(HE)_3_-Z_IGF-1R:4551_ (**B**) binding to ÌGF-1R-expressing cells in vitro. The data are presented as average ± standard deviation of three samples. The cells were incubated with 1 nM solution of the radiolabeled conjugates. For blocking, receptors were saturated with 1000-fold molar excess of non-labeled conjugate. Binding to blocked cells was significantly (*p* < 0.05) lower compared with non-blocked cells.

The results of the qualitative receptor saturation experiment are presented in Figure 4. The blocking of IGF-1R with an excess of non-labeled NODAGA-(HE)_3_-Z_IGF-1R:4551_ resulted in significantly (*p* < 0.05) lower binding of both [^68^Ga]Ga-NODAGA-(HE)_3_-Z_IGF-1R:4551_ and [^111^In]In-NODAGA-(HE)_3_-Z_IGF-1R:4551_ in blocked groups than in the non-blocked groups for both cell lines. This demonstrated that the binding was saturable.

**Figure 5:**
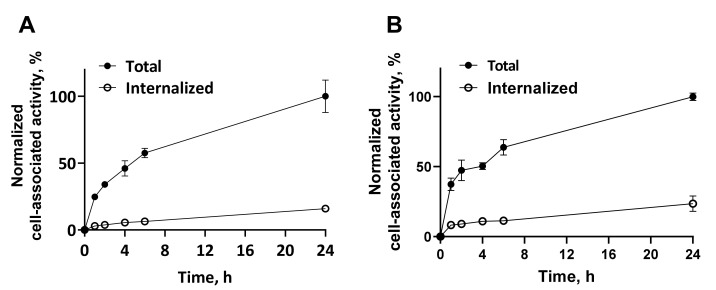
Normalized cellular processing of [^111^In]In-NODAGA-(HE)_3_-Z_IGF-1R:4551_ after binding to IGF-1R-expressing DU145 (**A**) and SKOV3 (**B**) cells in vitro. The data are presented as the average ± standard deviation of three samples.

Data concerning the binding and internalization of [^111^In]In-NODAGA-(HE)_3_-Z_IGF-1R:4551_ in IGF-1R-expressing DU145 and SKOV3 cell lines are presented in Figure 5. The pattern of binding to both cell lines was similar. An initial rapid increase of cell-associated activity was recorded, followed by slower increase. The internalization rate by both cell lines was rather slow. The internalized fraction 24 h after the start of incubation was 15.9 ± 1.2% and 23.5 ± 5.5% of the cell-associated activity for DU145 and SKOV3 cells, respectively.

### 3.4. In Vivo Evaluation

**Figure 6:**
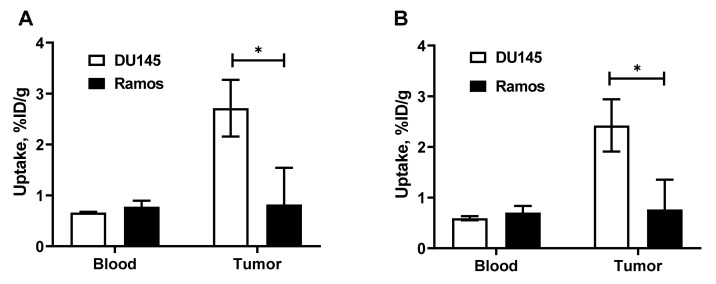
In vivo specificity: accumulation of [^111^In]In-NODAGA-(HE)_3_-Z_IGF-1R:4551_ (**A**) and [^68^Ga]Ga-NODAGA-(HE)_3_-Z_IGF-1R:4551_ (**B**) in IGF-1R-positive DU145 and IGF-1R-negative Ramos xenografts. Asterisk (*) marks a significant difference (*p* < 0.05) between uptake in DU145 and Ramos xenografts.

A comparison of [^68^Ga]Ga-NODAGA-(HE)_3_-Z_IGF-1R:4551_ and ^111^In-labeled NODAGA-(HE)_3_-Z_IGF-1R:4551_ uptake in IGF-1R-positive DU145 and IGF-1R-negative Ramos xenografts at 1 h is presented in Figure 6. The uptake in IGF-1R-negative Ramos xenografts was significantly (*p* < 0.05) lower than in IGF-1R-positive xenografts. There was no significant difference (*p* > 0.05) between the concentration of the tracers in blood.

The biodistribution results of [^111^In]In-NODAGA-(HE)_3_-Z_IGF-1R:4551_ and [^68^Ga]Ga-NODAGA-(HE)_3_-Z_IGF-1R:4551_ in BALB/c *nu/nu* mice bearing IGF-1R-positive DU145 xenografts are presented in Table 2. Both tracers were characterized by a rapid clearance from blood (blood concentration at 1 h p.i. was 0.6 ± 0.04 and 0.66 ± 0.02% ID/g for [^68^Ga]Ga-NODAGA-(HE)_3_-Z_IGF-1R:4551_ and [^111^In]In-NODAGA-(HE)_3_-Z_IGF-1R:4551_, respectively. The blood concentration was further decreased with time, although at a slower rate. Another distinguished feature of both tracers was a high renal uptake. One hour after injection, the kidneys retained 67 ± 4% of the injected ^68^Ga activity (uptake 250 ± 1% ID/g) and 62 ± 4% of the injected ^111^In activity (uptake 231 ± 7% ID/g). The renal uptake was constant over time for both nuclides (no significant difference, *p* > 0.05, between the time points). A noticeable uptake of both tracers was also found in the lung, liver, stomach, and colon. There was an apparent radionuclide-based difference in the biodistribution. The uptake of [^111^In]In-NODAGA-(HE)_3_-Z_IGF-1R:4551_ was significantly (*p* < 0.05 in a paired *t*-test) higher in the salivary gland, lung, pancreas, and colon at 1 h after injection, compared to the uptake of [^68^Ga]Ga-NODAGA-(HE)_3_-Z_IGF-1R:4551_.

The tumor uptake of both tracers remained stable between 1 and 3 h after injection (no significant difference between time points), but the uptake of [^111^In]In-NODAGA-(HE)_3_-Z_IGF-1R:4551_ decreased significantly (1.4-fold) by 24 h. There was a decrease of the uptake in normal organs and tissues between 1 and 3 h after injection, but it was less that 2-fold. Accordingly, only the increase of tumor-to-blood ratio between these time points was prominent for both tracers (*p* < 0.001), and the increase of tumor-to-muscle ratio was significant (*p* < 0.05) (Table 3). Increasing the time between injection and measurement for [^111^In]In-NODAGA-(HE)_3_-Z_IGF-1R:4551_ from 1 to 24 h resulted in a significant increase of tumor-to-organ ratios, but the increase was mainly (except from blood) less than two-fold.

**Figure 7:**
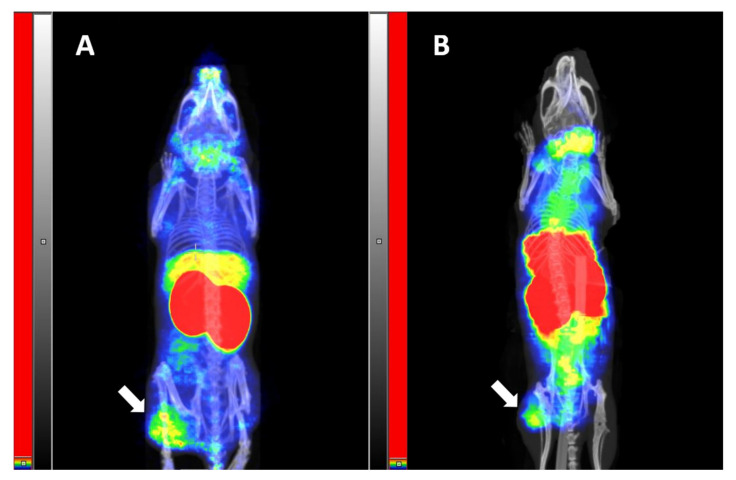
Imaging of IGF-1R expression in DU145 xenografts, using [^68^Ga]Ga-NODAGA-(HE)_3_-Z_IGF-1R:4551_ (**A**) and [^111^In]In-NODAGA-(HE)_3_-Z_IGF-1R:4551_ (**B**). The images were acquired 1 h after injection, using nanoScan PET/CT (**A**) and nanoScan SPECT/CT (**B**), and presented as maximum intensity projections. The arrows point at the tumors.

Images of mice bearing IGF-1R-expresing DU145 xenografts are presented in Figure 7. Both tracers, [^68^Ga]Ga-NODAGA-(HE)_3_-Z_IGF-1R:4551_ (Figure 7A) and [^111^In]In-NODAGA-(HE)_3_-Z_IGF-1R:4551_ (Figure 7B), were capable of visualization of the tumors. Besides the tumors, a high activity uptake was observed in the kidneys, livers, and salivary glands, and this result is in agreement with the biodistribution data. Overall, the PET imaging provided better tumor visualization compared to the SPECT imaging.

## 4. Discussion

Excellent imaging of the expression level of several cancer-associated molecular targets by using radiolabeled affibody molecules has been demonstrated in preclinical studies and in clinical trials [38]. The small size of these targeting proteins facilitates their prompt localization in tumors on the one hand, and a rapid clearance of an unbound tracer from blood on the other hand, enabling high contrast of imaging within a couple of hours after injection. Furthermore, a precondition for the high contrast imaging is a strong affinity of the affibody probe to the molecular target [53], and affibody molecules with strong sub-picomolar affinity to many different cancer-relevant targets have been identified [39]. The typical ability of affibody molecules to refold after denaturation permits the use of elevated temperatures and a broad range of pH values during labeling, thus expanding the repertoire of suitable labeling methods compared to those available to most mAbs.

An important feature of affibody molecules to consider is the strong influence of radionuclide and a chelator for its attachment on their biodistribution and targeting characteristics [38]. This is a double-edged sword: Selection of an unfortunate combination might result in a tracer with poor imaging properties. Conversely, a systematic optimization of the molecular design (including labeling chemistry, i.e., the selection of radionuclide, chelator, and position of the label) of the affibody-based tracer may appreciably increase the imaging contrast and sensitivity of the procedure [38]. This is particularly important in the case of imaging of IGF-1R, which usually only has a modest expression in tumors, accompanied by a noticeable expression in normal tissues.

Our previous imaging experience with HER3 expression using affibody molecules [54] showed that placement of a (HE)_3_-tag at the N-terminus and a NODAGA-chelator at the C-terminus of the affibody provided the best PET imaging when ^68^Ga was used as a label. However, the compositions of amino acids of the binding surface of HER3- and IGF-1R-binding affibody molecules are different, and this will likely influence the biodistribution profile of the tracers appreciably by contribution to off-target interactions in vivo [55,56,57]. Thus, the selection of an optimal molecular design should be performed for every new affibody-based imaging probe.

The results of this study confirmed the favorable features of NODAGA-(HE)_3_-Z_IGF-1R:4551_ for imaging of IGF-1R expression. The protein was efficiently labeled with both ^68^Ga and ^111^In, and the labels were stable (Table 1). Generally, affibody molecules are stable to proteolysis [38]. Particularly, earlier studies have demonstrated that Z_IGF-1R:4551_ is stable in murine serum for at least one hour [41,43], and affibody molecules are nearly completely cleared from blood by one hour after injection. High thermodynamic stability and kinetic inertness of NODAGA complexes with ^68^Ga and ^111^In have also been demonstrated earlier [49]. Still, there is always a possibility that a fraction of a radionuclide would be coupled not by a macrocyclic chelator but by an unspecific “chelating pocket”, which is formed by electron-donating sidechains. There is a risk that this coupling would be unstable and result in a release of a radionuclide in vivo. Therefore, we challenged the radiolabeled affibody molecules with a 1000-fold excess of EDTA, which should remove nuclides from weak binding sites. The results of this challenge (Table 1) suggest stable coupling of radionuclides to NODAGA-(HE)_3_-Z_IGF-1R:4551_. The radio-HPLC chromatograms (Figure 3) did not reveal any degradation of the proteins during labeling. Although the labeling conditions (temperature up to 60 °C, pH 3.6) will cause irreversible denaturation of most antibodies, both [^68^Ga]Ga-NODAGA-(HE)_3_-Z_IGF-1R:4551_ and [^111^In]In-NODAGA-(HE)_3_-Z_IGF-1R:4551_ demonstrated specific binding to IGF-1R-expressing cell lines in vitro and in vivo after labeling (Figure 4). It has to be noted that, due to low level of IGF-1R expression (B_max_ = 3.6 × 10^4^ receptors per cell for the cells line DU145 with the highest expression, DU-145 [41]), the absolute values of the target-bound activity were modest. The internalization of radiolabeled NODAGA-(HE)_3_-Z_IGF-1R:4551_ was quite slow, with less than 30% after 24 h incubation (Figure 5). This pattern is typical for other IGF-1R binding affibody molecules (see, for example, Reference [43]). Accumulation of both [^68^Ga]Ga-NODAGA-(HE)_3_-Z_IGF-1R:4551_ and [^111^In]In-NODAGA-(HE)_3_-Z_IGF-1R:4551_ in the IGF-1R-positive DU145 xenografts was significantly (*p* < 0.05) higher than in the IGF-1R-negative Ramos xenografts (Figure 6), thus suggesting IGF-1R-specific uptake in tumors in vivo. Experimental imaging (Figure 7) confirmed that the visualization of IGF-1R expression in tumors is possible by using both PET and SPECT already 1 h after injection.

The half-life of ^68^Ga (T_1/2_ = 67 min) permits imaging up to 3–4 h after injection. Our data suggest that an increase of the time between injection and imaging, from 1 to 3 h, would increase the tumor-to-blood ratio by approximately two-fold (Table 3), which can be expected to further improve the imaging contrast. ^111^In is a more long-lived (T_1/2_ = 2.8 d) nuclide than ^68^Ga, which permits image acquisition at later time points. Extending the time from 1 h to 24 h would permit an increase of the tumor-to-blood ratio from 4.1 ± 0.8 to 25.1 ± 1.3. It has to be noted that the uptake in lung, pancreas, stomach, colon, and salivary gland is IGF-1R-specific [40,41,43], and the clearance from these tissues was slow. The increase of ratios of radioactivity concentrations in tumors and these organs with time was only modest.

To evaluate the effect of the molecular design on the imaging properties of the anti-IGR-1R affibody imaging probes, we summarized their tumor-to-blood ratios in Table 4. Since the bloodborne activity contributes to the background in any organ and tissue, the tumor-to-blood ratio is one of most universal characteristics for comparison of different imaging probes. Almost all tracers (except for [^64^Cu]Cu-NOTA-Z_IGF-1R:4:40_) were evaluated in the same in vivo model, which facilitated a relatively fair comparison. It is obvious that already 3 h after injection [^68^Ga]Ga-NODAGA-(HE)_3_-Z_IGF-1R:4551_ provides as tumor-to-blood ratio as [^99m^Tc]Tc-Z_IGF-1R:4551-GGGC_ at 8 h after injection. [^68^Ga]Ga-NODAGA-(HE)_3_-Z_IGF-1R:4551_ also exceeds the tumor-to-blood ratio provided by other earlier developed affibody-based tracers in the same animal model. A comparison with other imaging probes is more complicated because different animal models were used, the affinity of the probes for IGF-1R differs, and their composition and molecular sizes are different. However, a comparison may still provide some context for [^68^Ga]Ga-NODAGA-(HE)_3_-Z_IGF-1R:4551_ and [^111^In]In-NODAGA-(HE)_3_-Z_IGF-1R:4551_. The tumor-to-blood ratio provided by [^68^Ga]Ga-NODAGA-(HE)_3_-Z_IGF-1R:4551_ is higher than the ratio provided by [^111^In]In-IGF-1(E3R) at 4 h after injection (5.8) [37]. It is as high as the value provided by ^111^In-labeled intact antibody R1507 at 7 days after injection (8.1 ± 2.5) or by its F(ab’)_2_ fragment at 24 h after injection (7.7) [32]. When PET is not available, ^111^In could be used as a label for NODAGA-(HE)_3_-Z_IGF-1R:4551_. The disadvantages of the SPECT detection might be alleviated by an increase of the interval between injection and imaging to 24 h. At this time point, [^111^In]In-NODAGA-(HE)_3_-Z_IGF-1R:4551_ provides the highest tumor-to-blood ratio reported for any of the IGR-1R imaging probes reported on in the literature. Obviously, ^99m^Tc has more favorable imaging properties and is cheaper than ^111^In. However, the development of imaging probes is very expensive, and it is not realistic to expect that two different tracers with different chelators, one for PET and one for SPECT, would be developed simultaneously. The development of NODAGA-(HE)_3_-Z_IGF-1R:4551_, which might be used for both radionuclide imaging modalities, seems to be a more realistic choice.

In conclusion, a molecular design of the Z_IGF-1R:4551_ affibody molecule that includes the placement of the (HE)_3_-tag at N-terminus and site-specific coupling of a NODAGA chelator at C-terminus provides a tracer with improved imaging properties for the visualization of IGF-1R expression in malignant tumors when using PET and SPECT modalities.

## Figures and Tables

**Table 1:** Stability of radiolabeled conjugates under challenge with 1000-fold molar excess of EDTA. The challenge time was 1 h for ^68^Ga and 6 h for ^111^In. The data are presented as an average (*n* = 2) ± maximum error.

	Protein-Associated Activity	
	[^68^Ga]Ga-NODAGA-(HE)_3_-Z_IGF-1R:4551_	[^111^In]In-NODAGA-(HE)_3_-Z_IGF-1R:4551_
1000-fold molar excess EDTA	98.4 ± 0.2%	98.2 ± 0.5%
Control	98.3 ± 0.2%	99.4 ± 0.2%

**Table 2:** Biodistribution of [^68^Ga]Ga-NODAGA-(HE)_3_-Z_IGF-1R:4551_ and [^111^In]In-NODAGA-(HE)_3_-Z_IGF-1R:4551_ in BALB/C *nu/nu* mice bearing DU145 xenografts.

	[^68^Ga]Ga-NODAGA-(HE)_3_-Z_IGF-1R:4551_		[^111^In]In-NODAGA-(HE)_3_-Z_IGF-1R:4551_		
	1 h	3 h	1 h	3 h	24 h
Blood	0.6 ± 0.04	0.31 ± 0.01 ^b^	0.66 ± 0.02	0.36 ± 0.02 ^b^	0.077 ± 0.004
Salivary gland	2.99 ± 0.03 ^a^	2.3 ± 0.4	3.7 ± 0.2 ^a^	3 ± 0.3	1.47 ± 0.04
Lung	3.9 ± 0.1 ^a^	3.2 ± 0.2	4.19 ± 0.02 ^a^	3.8 ± 0.2	1.8 ± 0.2
Liver	3.2 ± 0.1 ^a^	2.7 ± 0.1 ^b^	3.7 ± 0.1 ^a^	3.4 ± 0.1 ^b^	2 ± 0.2
Spleen	1.8 ± 0.1	1.5 ± 0.2	2.1 ± 0.1	2 ± 0.2	1.3 ± 0.2
Pancreas	2.3 ± 0.1 ^a^	1.7 ± 0.4	2.65 ± 0.04 ^a^	2.1 ± 0.5	1.5 ± 0.1
Stomach	2.6 ± 0.1	2.1 ± 0.1 ^b^	3.2 ± 0.2	2.6 ± 0.1 ^b^	1.4 ± 0.2
Colon	2.94 ± 0.03 ^a^	2.4 ± 0.2 ^b^	3.4 ± 0.1 ^a^	3.2 ± 0.2 ^b^	1.5 ± 0.2
Kidney	250 ± 11	275 ± 25	231 ± 7	258 ± 26	246 ± 23
Tumor	2.4 ± 0.5	2.4 ± 0.1 ^b^	2.7 ± 0.6	2.8 ± 0.2 ^b^	1.9 ± 0.1
Muscle	0.45 ± 0.03	0.31 ± 0.04	0.54 ± 0.02	0.4 ± 0.1	0.2 ± 0.03
Bone	0.8 ± 0.1	0.7 ± 0.1	1 ± 0.1	0.9 ± 0.2	0.5 ± 0.1
GI *	3 ± 0.1 ^a^	2.6 ± 0.2 ^b^	3.5 ± 0.1 ^a^	3.2 ± 0.2 ^b^	1.7 ± 0.2
Carcass *	13.2 ± 0.2 ^a^	9.6 ± 0.7 ^b^	15.9 ± 0.4 ^a^	13.3 ± 1.5 ^b^	6.2 ± 0.6

* Data for gastrointestinal tract (GI) are presented as %ID per whole sample with content. Data for carcass are presented as %ID for whole sample. The data are presented as average (*n* = 4) values ± SD. ^a^ Significant difference (*p* < 0.05 in paired *t*-test) between [^68^Ga]Ga-NODAGA-(HE)_3_-Z_IGF-1R:4551_ and [^111^In]In-NODAGA-(HE)_3_-Z_IGF-1R:4551_ at 1 h p.i. ^b^ Significant difference (*p* < 0.05 in paired *t*-test) between [^68^Ga]Ga-NODAGA-(HE)_3_-Z_IGF-1R:4551_ and [^111^In]In-NODAGA-(HE)_3_-Z_IGF-1R:4551_ at 3 h p.i.

**Table 3:** Tumor-to-organ ratios for [^68^Ga]Ga-NODAGA-(HE)_3_-Z_IGF-1R:4551_ and [^111^In]In-NODAGA-(HE)_3_-Z_IGF-1R:4551_ in BALB/C *nu*/*nu* mice bearing DU145 xenografts.

	[^68^Ga]Ga-NODAGA-(HE)_3_-Z_IGF-1R:4551_		[^111^In]In-NODAGA-(HE)_3_-Z_IGF-1R:4551_		
	1 h	3 h	1 h	3 h	24 h
Blood	4 ± 0.7	7.8 ± 0.2	4.1 ± 0.8	8 ± 0.6	25.1 ± 1.3
Salivary gland	0.8 ± 0.2	1.1 ± 0.1	0.7 ± 0.2	1 ± 0.1	1.3 ± 0.1
Lung	0.6 ± 0.1	0.8 ± 0.1	0.6 ± 0.1	0.74 ± 0.03	1.1 ± 0.1
Liver	0.8 ± 0.1	0.91 ± 0.02	0.7 ± 0.1	0.84 ± 0.04	1 ± 0.1
Spleen	1.4 ± 0.3	1.6 ± 0.1	1.3 ± 0.3	1.5 ± 0.2	1.6 ± 0.2
Pancreas	1.1 ± 0.2	1.5 ± 0.4	1 ± 0.2	1.4 ± 0.4	1.3 ± 0.1
Stomach	0.9 ± 0.2	1.14 ± 0.03	0.9 ± 0.1	1.1 ± 0.1	1.4 ± 0.1
Colon	0.8 ± 0.2	1 ± 0.1	0.8 ± 0.2	0.9 ± 0.1	1.3 ± 0.1
Kidney	0.010 ± 0.002	0.009 ± 0.001	0.012 ± 0.002	0.011 ± 0.002	0.008 ± 0.001
Muscle	5.3 ± 0.9	8 ± 1	5 ± 1	7.6 ± 1.1	10 ± 1.6
Bone	3 ± 0.9	3.5 ± 0.6	2.7 ± 0.8	3.2 ± 0.7	4 ± 0.7

**Table 4:** Tumor-to-blood ratios provided by different radiolabeled variants of Z_IGF-1R:4551_ in DU145 prostate cancer xenografts in mice.

	Tumor-to-Blood Ratio					
	[^111^In]In-DOTA-H_6_-Z_IGF-1R:4551_ [40]	[^99m^Tc]Tc (CO)_3_-(HE)_3_-Z_IGF-1R:4551_ [41]	[^99m^Tc]Tc-Z_IGF-1R:4551_-GGGC [43]	[^64^Cu]Cu-NOTA-Z_IGF-1R:4:40_ * [44]	[^68^Ga]Ga-NODAGA-(HE)_3_-Z_IGF-1R:4551_ (This Study)	[^111^In]In-NODAGA-(HE)_3_-Z_IGF-1R:4551_ (This Study)
1	1.3 ± 0.2		3.1 ± 0.3		4.1 ± 0.7	4.1 ± 0.8
3					7.8 ± 0.2	8.0 ± 0.6
4	2.5 ± 0.2	3.5 ± 0.7	6.2 ± 0.9			
8	3.3 ± 0.2	4.4 ± 0.3	7.6 ± 2.3			
24	5.1 ± 0.3	5.4 ± 0.4		4.1 ± 0.6		25.1 ± 1.3

* Data are for U87MG xenografts.

## Data Availability

All data are contained within the manuscript.
